# Bone‐Mimetic Osteon Microtopographies on Poly‐ε‐Caprolactone Enhance the Osteogenic Potential of Human Mesenchymal Stem Cells

**DOI:** 10.1002/mabi.202400311

**Published:** 2024-09-05

**Authors:** Matthias Vostatek, Elettra Verin, Marvin Tamm, Mario Rothbauer, Stefan Toegel, Francesco Moscato

**Affiliations:** ^1^ Center for Medical Physics and Biomedical Engineering Medical University of Vienna Waehringer Guertel 18–20/4L Vienna 1090 Austria; ^2^ Austrian Cluster for Tissue Regeneration Donaueschingenstrasse 13 Vienna 1200 Austria; ^3^ Karl Chiari Lab for Orthopedic Biology Department of Orthopedics and Trauma Surgery Medical University of Vienna Waehringer Guertel 18–20 Vienna 1090 Austria; ^4^ Faculty of Technical Chemistry Technische Universitaet Wien Getreidemarkt 9 Vienna 1060 Austria; ^5^ Ludwig Boltzmann Institute for Arthritis and Rehabilitation Spitalgasse 23/BT88 Vienna 1090 Austria; ^6^ Ludwig Boltzmann Institute for Cardiovascular Research Waehringer Guertel 18–20/4L Vienna 1090 Austria

**Keywords:** biomimetic, microtpography, osseointegration, two‐photon polymerization

## Abstract

The attributes of implant surfaces are pivotal for successful osseointegration. Among surface engineering strategies, microtopography stands out as a promising approach to promote early cellular interactions. This study aims to design and craft a novel biomimetic osteon‐like surface modification and to compare its impact on human mesenchymal stem cells (hMSCs) with four established topographies: blank, inverted pyramids, protrusions, and grooves. Poly‐ε‐caprolactone samples are fabricated using 2‐photon‐polymerization and soft lithography, prior to analysis via scanning electron microscopy (SEM), water contact angle (WCA), and protein adsorption assays. Additionally, cellular responses including cell attachment, proliferation, morphology, cytoskeletal organization, and osteogenic differentiation potential are evaluated. SEM confirms the successful fabrication of microtopographies, with minimal effect on WCA and protein adsorption. Cell attachment experiments demonstrate a significant increase on the osteon‐like structure, being three times higher than on the blank. Proliferation assays indicate a fourfold increase with osteon‐like microtopography compared to the blank, while ALP activity is notably elevated with osteon‐like microtopography at days 7 (threefold increase over blank) and 14 (fivefold increase over blank). In conclusion, the novel biomimetic osteon‐like structure demonstrates favorable responses from hMSCs, suggesting potential for promoting successful implant integration in vivo.

## Graphical abstract

The implant surface is crucial for osseointegration, with microtopography as a promising tool. This study designs a novel biomimetic osteon‐like surface and compares its impact on human mesenchymal stem cells with four topographies. Osteon‐like structure significantly enhances cell attachment, proliferation, and osteogenic differentiation. Combined with other techniques like nanoimprint lithography, osteon‐like microtopography has the potential to be used for future bone implants.

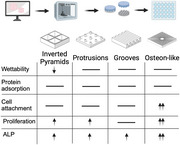

## 1 Introduction

Cell adhesion is a fundamental process for anchorage‐dependent cell survival within the surrounding matrix. It supports various cellular functions like migration, proliferation, and differentiation, while also initiating communication between cells and the environment, crucial for tissue development.^[^ ^1^, ^2^, ^3^, ^4^ ^]^ In fields like tissue engineering and regenerative medicine, artificial surfaces mimic natural matrix properties, regulating cell adhesion behavior.^[^ ^3^, ^5^ ^]^ Understanding cell behavior on surfaces is crucial for material and life sciences, particularly in biomedical and tissue engineering, to understand and anticipate potential biocompatibility issues and functional aspects.^[^ ^6^, ^7^ ^]^ In regenerative medicine, the restoration of bone defects or injuries remains a significant challenge. Consequently, research focuses on biomaterials for bone tissue engineering applications that meet structural, mechanical, and biological requirements crucial for effective bone regeneration.^[^ ^8^, ^9^, ^10^ ^]^

Osseointegration, a term introduced by Per‐Ingvar Brånemark in 1976, defines the direct connection between living bone and the surface of an implant, with the term now extending to biomaterials capable of osseointegration.^[^ ^11^, ^12^ ^]^ The osseointegration process unfolds through three phases: osteoconduction, de novo bone formation, and bone remodeling.^[^ ^12^ ^]^ Osteoconduction initiates new bone formation as osteogenic cells migrate to the implant surface.^[^ ^13^ ^]^ At the interface, de novo bone formation involves sequential reactions including cell binding, chemotaxis, and differentiation.^[^ ^14^ ^]^ The interplay between initial cell recruitment and bone formation underscores the significance of implant surfaces and biological factors.^[^ ^15^ ^]^ Despite the presence of established bone implants, implant failure persists due to inadequate osseointegration leading to implant loosening, prompting efforts to develop novel implant materials.^[^ ^16^, ^17^, ^18^, ^19^, ^20^ ^]^

Hence, the surface properties of implants, including chemical, biochemical, and biophysical aspects, play a crucial role in influencing interactions between cells and bone implants, consequently impacting cellular responses both in vivo and in vitro.^[^ ^15^, ^21^, ^22^ ^]^ Comprehending the interplay between biomaterials and cells is crucial, as it directly influences cell proliferation, differentiation, migration, and the integration of biomaterials into host tissue. Various surface treatments enhance biological surface properties, promoting osseointegration by facilitating stronger and faster bone formation, and improving stability.^[^ ^23^, ^24^, ^25^, ^26^ ^]^

**Figure 1:**
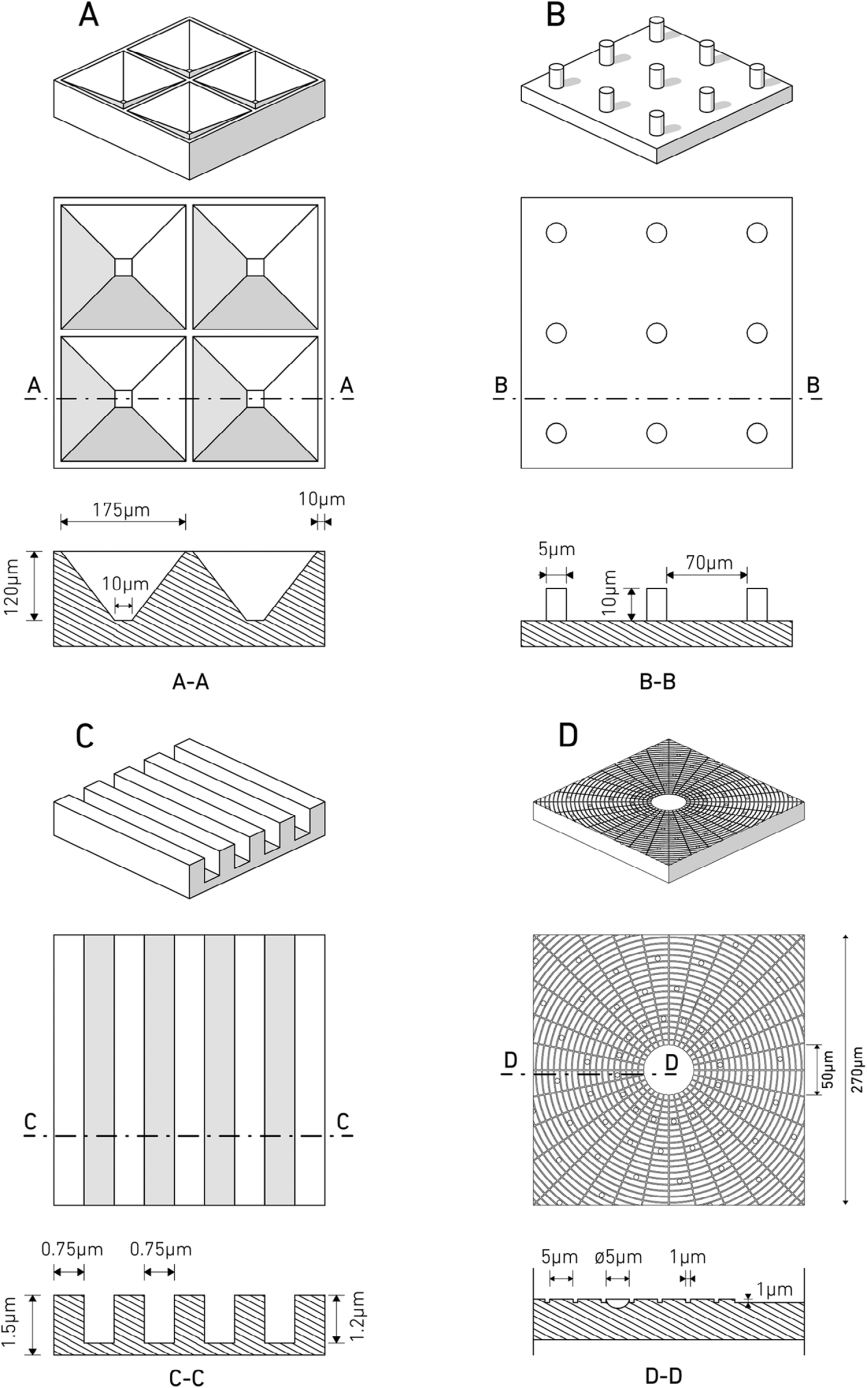
Microtopographies. Inverted pyramids A) microtapered pits, inverted pyramid with cutoff tip, with depth = 120 µm, pitch = 185 µm, width at top = 175 µm, width at bottom = 10 µm, ridge = 10 µm. Protrusions. B) Cylinders D = 5 µm H = 10 µm spacing = 70 µm. Grooves C) grooves with width = 0.75 µm, pitch = 1.5 µm, depth = 1.3 µm. Osteon‐Like D). Panels are not drawn in the same scale.

Acknowledging the profound impact of cells’ sensitivity to their microenvironment, recent advancements in miniaturization techniques, such as two‐photon polymerization printing, have deepened our understanding of the influence of surface microtopography.^[^ ^27^, ^28^ ^]^ These effects encompass alterations in cell adhesion, migration, cytoskeletal organization, genome regulation, and differentiation.^[^ ^29^, ^30^, ^31^, ^32^, ^33^ ^]^ In the field of biomaterials, surface morphology plays a critical role in influencing cell behavior, particularly in the context of bone implants. The selection of surface microtopographies for this study was based on a comprehensive review of existing literature and their proven benefits in enhancing osteogenic outcomes. Specifically, four types of surface morphologies were chosen: inverted pyramids, protrusions, grooves, and an osteon‐like structure. The microstructure here called “inverted pyramids” was first described by Hamilton, Chehroudi et al. as microfabricated tapered pits with increased bone mineralization on titanium.^[^ ^34^ ^]^ Cylindrical protrusions, identified in a paper by Nosonovsky and Bhushan, exhibit improved wettability properties on silica, leading to increased hydrophilicity and therefore improved cell attachment.^[^ ^35^ ^]^ Grooves reported by Cipriano et al. were associated with increased cell attachment and spreading on titanium.^[^ ^36^ ^]^ The osteon‐like pattern, a biomimetic microtopography, combines features of these structures and is modeled after osteons found in bone tissue, using Ardizzoni's light microscope micrographs and scanning electron microscopy (SEM) images.^[^ ^37^ ^]^ This biomimicking structure includes grooves with a width of 1 µm and an interspacing of 5 µm forming concentric circles, modeling the concentric lamellae interspaced with canaliculi. Additionally, it features half‐sphere pits with a diameter of 5 µm resembling lacunae, which house osteocytes in natural bone. Sketches of all microtopographies are displayed in **Figure** **1**. These microstructures were selected due to their demonstrated advantages over non‐structured surfaces, including improved bone mineralization, enhanced wettability, increased cell attachment, and their biomimetic properties. Additionally, these structures were chosen for their manufacturability using advanced 3D printing techniques, particularly the UpNano nanoprinter, ensuring precise fabrication.

This study aims to compare the pro‐osteogenic potential of newly established osteon‐like structures mimicking natural bone microstructure to four well‐tested surface microtopographies. These microtopographies include grooves, protrusions and pyramids. The goal is to provide an implant surface that provides a more natural bone‐cell niche to ameliorate human mesenchymal stem cells differentiation (hMSCs). This enhancement was confirmed by assessing cell attachment, proliferation, cytoskeletal organization, and alkaline phosphatase (ALP) activity.

## 2 Results and Discussion

**Figure 2:**
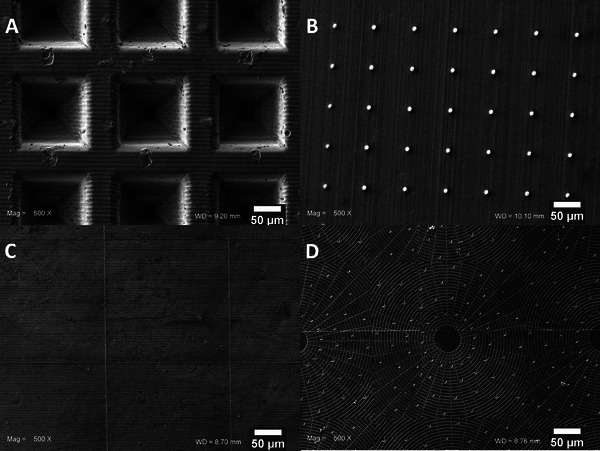
SEM images of inverted pyramids A), protrusions B), grooves C), and osteon‐like structure D) after gold sputtering. Magnification of 500x, Scale bar is 50 µm.

In this study, four different microtopographies were created on Poly‐ε‐caprolactone (PCL) (inverted pyramids, protrusions, grooves, and a newly developed biomimicking osteon‐like structure), alongside a non‐structured PCL surface serving as a control. Morphological evaluation of the microstructured PCL surfaces is shown in **Figure** **2**. The microstructures, fabricated through printing and soft lithography, were effectively retained during the molding process to obtain the final PCL samples.

These were investigated for their pro‐osteogenic potential by assessing wettability, protein adsorption, cell attachment, proliferation, cytoskeletal organization, and ALP activity.

The long‐term success of an implant is heavily reliant on osseointegration, a process significantly influenced by the interaction between the implant surface and cells.^[^ ^38^, ^39^ ^]^ Initially, preosteogenic precursor cells are recruited to the implant site, where they must proliferate and differentiate into osteogenic lineages. This differentiation is crucial for bridging the gap between the implant and bone.^[^ ^40^ ^]^ Therefore, evaluating stem cell adhesion, proliferation, and differentiation potential on engineered surfaces is vital for understanding how different surface topographies influence the osseointegration of implants.^[^ ^41^ ^]^

**Figure 3:**
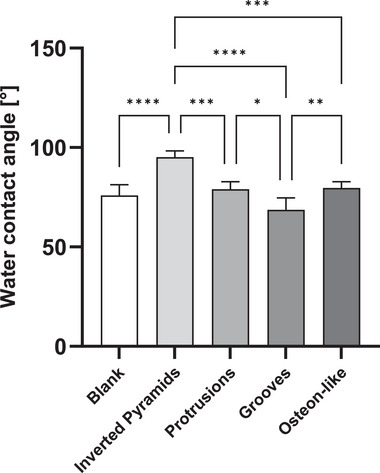
Contact angles of water on blank, inverted pyramids, protrusions, grooves, and osteon‐like structures measured with sessile drop method. Five discs were measured. P‐values smaller than 0.05 were considered statistically significant. ^*^ *p*‐value< 0.05; ^**^ *p*‐value< 0.01; ^***^ *p*‐value< 0.001; ^****^ *p*‐value< 0.0001.

Improving the wettability of the material is known to enhance protein adsorption and cell attachment.^[^ ^42^, ^43^, ^44^ ^]^ Therefore, the hydrophobicity of PCL discs with and without microtopographies was investigated with contact angle analyses. As represented in **Figure** **3**, the blank without microstructures, with a water contact angle (WCA) of 76.0°±5.3°, was taken as a negative control and reference for the PCL discs with microstructures. Protrusions with a WCA of 79.0°±3.8°; grooves with 68.7±5.9°; and osteon‐like with 79.7±3.2° exhibited no significant increase in WCA compared to the blank. However, inverted pyramids, with a WCA of 95.2±3.1°, showed a statistically significant increase in WCA compared to the blank, as well as to the other microtopographies tested. Hence, rendering the surface more hydrophobic and potentially reducing the likelihood for cell attachment.^[^ ^42^, ^43^, ^44^ ^]^

This phenomenon could be explained by the Cassie‐Baxter state, where air is trapped inside the pit structure, elevating the water contact angle of the surface.^[^ ^45^, ^46^ ^]^ While surface wettability and protein adsorption should theoretically correlate, no differences were observed in the protein adsorption values between the control and inverted pyramids.

**Figure 4:**
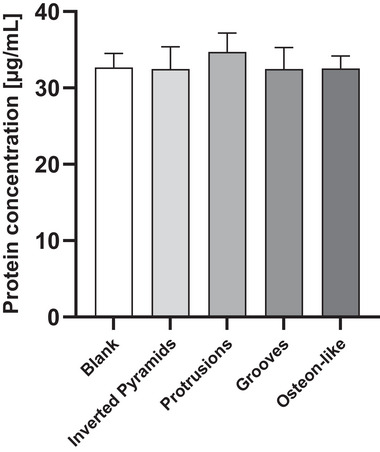
Protein adsorption on blank, inverted pyramids, protrusions, grooves, osteon‐like structures measured with Pierce Bradford protein assay kit displayed as mean ± standard deviation. Ten measurements of each microtopography were taken.

The amount of protein on PCL discs without microstructure (blank) was 32.69±1.83 µg mL^−1^, serving as a negative control and reference for microstructured PCL discs (**Figure** **4**). Inverted pyramids with a protein concentration of 32.49±2.88 µg mL^−1^, protrusions with 34.72±2.46 µg mL^−1^, grooves with 32.48±2.79 µg mL^−1^, and osteon‐like with 32.56±1.61 µg mL^−1^.

**Figure 5:**
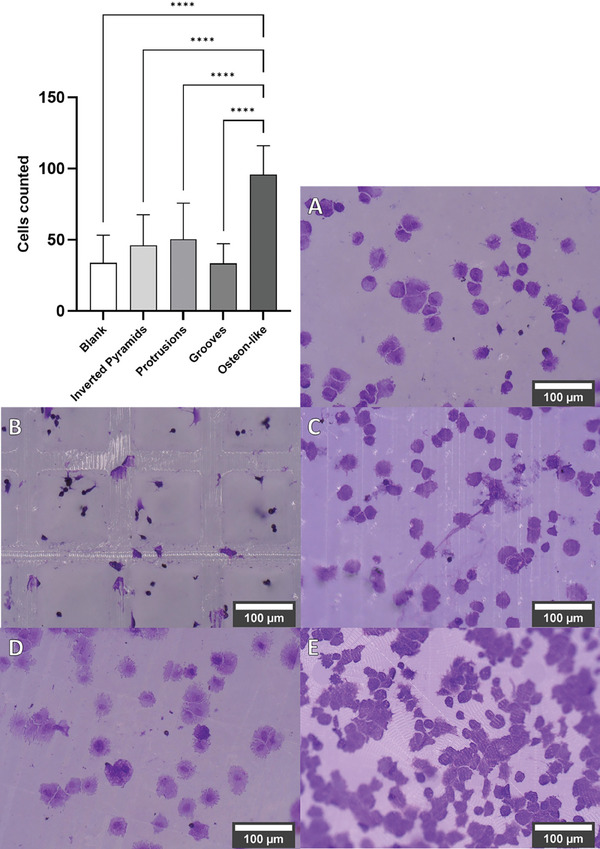
Comparison of the hMSC attachment on blank (A), inverted pyramids (B), protrusions (C), grooves (D), and osteon‐like (E) microstructures measured via crystal violet staining. The data represent the number of counted cells per image. ^****^ *p*‐values< 0.0001. *p*‐values< 0.05 were considered significant. Scale bars are 100 µm.

However, it's essential to note that the protein adsorption assay only quantifies the amount of protein attached to the surface, without providing information on the composition or conformation of the attached proteins. The type and conformation of proteins attached could significantly impact subsequent cell interactions.^[^ ^47^ ^]^ Surface topography plays a crucial role not only in driving cell attachment through wettability or protein adsorption but also by introducing additional micro‐patterns that can facilitate surface functionalization. These microtopographies serve as mechano‐physical cues and anchoring points to which cells can better adhere and spread, resulting in greater stability.^[^ ^48^, ^49^ ^]^ This was evident in the cell attachment assay, where the osteon‐like structure exhibited a significant improvement compared to the blank and other tested microtopographies. **Figure** **5** illustrates the outcome of the cell attachment assay, where five random image sections were analyzed for each of the five samples. On the unstructured blanks A), a mean cell count of 34±19 cells per image section was determined. On the inverted pyramids B), there were 33±13 cells per image section; on the protrusions C) 50±24 cells per image section; on the grooves D) 46±20 cells per image section; and on the osteon‐like E) 96±20 cells per image section.

**Figure 6:**
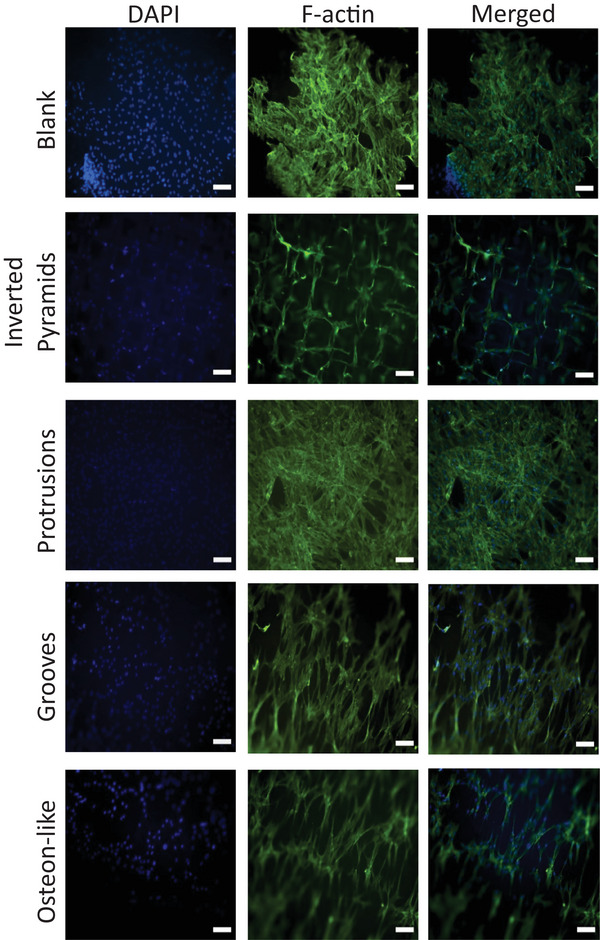
Immunoflourescent staining of hMSCs grown on blank, inverted pyramids, protrusions, grooves, and osteon‐like structures for 24 h. Cells were stained with DAPI (nuclei, blue), and F‐actin (green). The scale bars correspond to 100 µm.

Interestingly, the influence of mechano‐physical cues was also observed in the morphology of the cells on grooves, which changed into a unidirectional spreading of lamellipodia along the grooves of the microstructure, as displayed in **Figure** **6**. Cells seeded on the osteon‐like geometry were also elongated, similarly to the groove structure, indicating that the groove structures in the osteon‐like microtopography had an influence on spreading behaviors. While on the blank structure and on the protrusions the cells appeared to form a uniform and compact mass, on the inverted pyramids the cells elongated along the rims between single pyramids and migrated inside the structures. Due to the comparatively large size of the inverted pyramids, there is a significant distance between the bottom part and the rim of the pyramids, the focal plane depth allowed one to either focus on the upper or lower part of the microstructure. This means that one can either focus on the upper part or the lower part of the microstructure, which is why the two different images were needed for the inverted pyramids.

**Figure 7:**
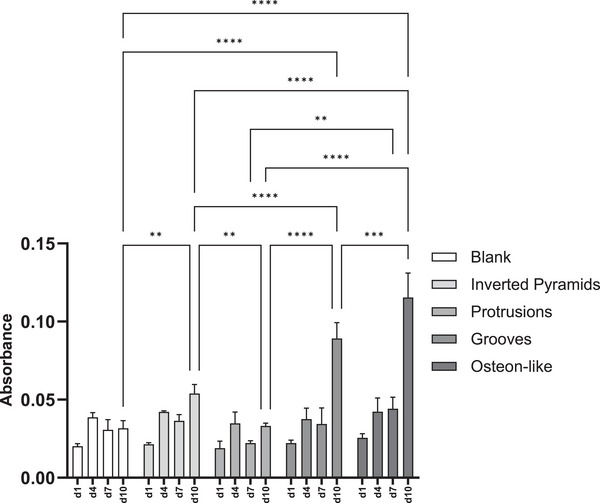
Comparison of the hMSCs proliferation on blank, inverted pyramids, protrusions, grooves, and osteon‐like microstructures measured via XTT assay at days 1, 4, 7, and 10. The data are given as absorbance measured and displayed as mean ± standard deviation. ^**^ *p*‐values< 0.01; ^***^ *p*‐values< 0.001; ^****^ *p*‐values< 0.0001.

Enhanced cell proliferation on the implant surface promotes quicker filling of the narrow gap between the implant and adjacent bone.^[^ ^40^ ^]^ This rapid closure facilitates accelerated osseointegration, effectively shortening the period required for the bone to integrate with the implant.^[^ ^40^ ^]^ Consequently, this leads to reduced recovery times for patients, enabling a swifter return to normal function. Therefore, an XTT assay was employed, which measures cell viability and proliferation via cellular metabolic activity. For most of the microstructured discs, but not for the control, the amount of viable cells tended to increase over time (**Figure** **7**). No significant differences between different microtopographies were observed at day 1, as well as at day 4. On day 7, the only significant difference can be seen in the difference between osteon‐like structures and protrusions. By day 10, the highest absorbance was found with the osteon‐like structure, followed by grooves and inverted pyramids, with significant differences compared to the blank and also to each other. While no additional assays were performed in this study, the robustness of the XTT assay is well‐documented and widely accepted for assessing cell proliferation and activity.^[^ ^50^ ^]^

**Figure 8:**
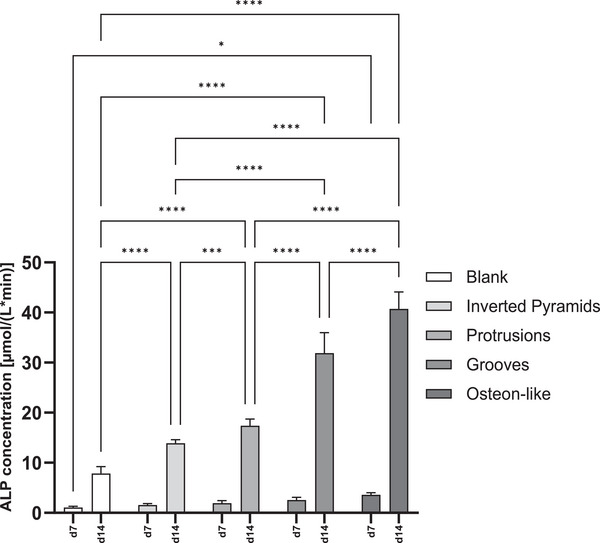
ALP activity of hMSCs differentiated with osteogenic differentiation medium into the osteogenic lineage on blank, inverted pyramids, protrusions, grooves, and osteon‐like structure. ALP activity was measured via Alkaline Phosphatase assay at day 7, and 14. The data are displayed as mean ± standard deviation for n = 5. ^*^ *p*‐values< 0.05; ^**^ *p*‐values< 0.01; ^***^ *p*‐values< 0.001; ^****^ *p*‐values< 0.0001. p‐values < 0.05 were considered significant.

Another important factor in osseointegration is not only the recruitment and proliferation of progenitor cells but also their differentiation.^[^ ^40^ ^]^ ALP has been consistently identified as a marker for osteogenic differentiation. Studies have shown that cells with osteoinductive potential, such as osteoblasts, exhibit high levels of ALP activity.^[^ ^51^ ^]^ Therefore, after differentiation into the osteogenic lineage, hMSCs’ ALP activity was quantified on days 7 and 14 after osteogenic induction. As shown in **Figure** **8**, for all samples the ALP activity increased over time. On day 7, all microstructured discs induced higher ALP activity than the negative control without microstructure, with the osteon‐like microtopography showing significantly higher activity (threefold increase over blank). On day 14, significant differences were found between all microstructures with the osteon‐like structure performing the best (fivefold increase over blank), followed by grooves (fourfold increase over blank), protrusions (twofold increase over blank), and inverted pyramids (1.7‐fold increase over blank), while the blank displayed the lowest level of ALP activity. It is worth noting that RT‐qPCR could provide a more comprehensive evaluation of osteogenic activity by verifying the expression of specific osteogenic genes, offering additional insights beyond what the ALP assay alone can reveal. Such investigations will thus require a dedicated follow‐up study.

Calcium deposits after differentiation, observed with Alizarin Red staining, are shown in supplementary information Figure S1 (Supporting Information). On day 7, the overview image (1x) suggests that the inverted pyramid structure has the most prominent calcium deposition, with little red staining on other substrates. However, closer inspection (500x) reveals calcium deposits on all microtopographies. By day 14, the inverted pyramids still show the most prominent calcium deposition with vibrant and evenly distributed red staining, followed by osteon‐like grooves.

Choosing PCL to create varied microtopographies in this study was advantageous due to its distinctive properties. PCL's biocompatibility minimizes adverse bodily reactions, making it well‐tolerated by the body without eliciting an adverse immune response.^[^ ^52^, ^53^ ^]^ Its biodegradability is key for scaffolds that need to be replaced by new bone tissue over time, as it slowly degrades into non‐toxic by‐products, reducing the need for secondary surgeries.^[^ ^54^, ^55^ ^]^ PCL's mechanical properties can be finely tuned to support bone regeneration effectively, offering the necessary flexibility and strength during the early stages of bone healing.^[^ ^56^ ^]^ Additionally, its low melting point (≈ 60 °C) makes PCL easy to mold into complex shapes, crucial for examining the impact of different surface textures.^[^ ^57^, ^58^ ^]^ Furthermore, PCL promotes cell attachment and proliferation, enhancing its suitability for bone tissue engineering, while also being cost‐effective.^[^ ^59^, ^60^ ^]^

While our study demonstrates the osteogenic potential of bone‐mimetic osteon microtopographies on PCL within the first two weeks, it is important to note that the biodegradable nature of PCL may compromise the long‐term stability of these microstructures. Although the microstructures remained intact within the two weeks of observation, literature suggests that significant PCL degradation, including molecular weight reduction and mass loss, may occur within several months.^[^ ^61^ ^]^ As degradation progresses, the defined architecture supporting osteogenesis may diminish, potentially reducing the sustained effectiveness of the microtopographies. This underscores the need for future research to explore the impact of PCL degradation on osteogenesis over extended periods and to develop strategies to maintain microstructural integrity for longer durations.

A limitation of this study is the absence of in vivo experimental data. While the in vitro findings offer valuable insights into the osteogenic potential of bone‐mimetic osteon microtopographies, their translation to in vivo conditions remains unconfirmed. Future research should include in vivo experiments to validate and extend the current findings.

The osteon‐like microtopography introduced in this study is particularly noteworthy, as it not only mimics the natural microstructure found in bone, making it inherently biomimetic, but also effectively combines elements of other microstructures commonly reported in the literature, such as grooves or pit‐like structures.^[^ ^62^, ^63^ ^]^

The enhancement observed in cell attachment, proliferation, and osteogenic differentiation on this osteon‐like surface represents a significant advancement in the development of osseointegrative implants. By closely replicating the intrinsic architectural features of bone, this microtopography facilitates a more natural interaction between the implant and surrounding bone tissue, thereby promoting critical early steps in osseointegration. This alignment of structural design with biological function is a critical step forward, underscoring the potential of such biomimetic surfaces to improve the integration and performance of orthopedic and dental implants in clinical settings.

## 3 Conclusion

The superior performance of bone‐mimetic osteon‐like surface patterning on bioresorbable implant interfaces, as confirmed by enhanced attachment, proliferation, and osteogenic differentiation of hMSCs compared to conventional surface patterns, indicates the potential for improving osseointegration in implants. This advancement holds promise for addressing challenges such as aseptic and septic loosening, which remain significant issues with traditional bone implants.^[^ ^64^ ^]^

For effective implementation of this bone‐mimetic osteon‐like microstructure on medical implants at scale, careful selection of appropriate technologies is essential. UV nanoimprint lithography emerges as a promising method due to its straightforward and cost‐effective nature, capable of replicating intricate micro‐ and nanostructures across large areas.^[^ ^65^ ^]^ Further validation through in vivo studies is necessary to verify the in vitro findings.

## 4 Experimental Section

### Mictrostructured PCL Design and Manufacturing

Four different microstructures were created to be investigated for their role in influencing pro‐osteogenic differentiation potential of MSCs. These microstructures include inverted pyramids, cylindrical protrusions, microgrooves and a novel osteon‐like geometry resembling native bone tissue, along with a blank, featureless surface used for control. The microstructures were designed as negatives with OpenSCAD and then printed in 2‐photon resin (Upbrix, UpNano GmbH, Vienna, Austria) via multiphoton lithography, using a two‐photon‐polymerization printer (UpNano GmbH, Vienna, Austria).

Starting from the UpBrix parts, silicone micropatterned molds were manufactured through a process of imprinting consisting of consecutive steps of molding and demolding of polydimethylsiloxane (PDMS, SYLGARD™ 184 Silicone Elastomer kit, Dow Corning, France), epoxy resin (Epoxy Resin Crystal Clear Kit, SigWong, China), PCL (molecular weight = 80.000, Sigma Aldrich, St. Louis, USA) and eventually PDMS.

PCL (Mw = 37.000, Polysciences, Inc., Warrington, USA) was melted into the obtained PDMS molds at 120 °C for 30 min, and consequently cured at −20 °C for 90 min.

### Morphological Evaluation of the Surface: SEM Imaging

After demolding, microstructured samples were coated with a 40 nm layer of gold using a sputter coater (Q150R ES, Quorum, UK) prior to imaging of their surface morphology with a SEM (EVO MA10, Zeiss, Germany).

### Surface Wettability: Water Contact Angle Measurement

The wettability properties of microstructures were assessed by measuring the contact angle of a water droplet on the respective microstructured surface, using a contact angle goniometer (DSA25E, Krüss, Hamburg, Germany) via the sessile drop method with a 2 µL distilled water droplet. The average contact angle and standard deviation were calculated based on measurements of five different discs for each microtopography as well as the non‐structured negative control.

### Protein Adsorption Assay

For assessing the concentration of proteins adhered to the surface, Pierce Bradford Protein assay kit (Thermo Scientific, ThermoFisher Scientific, Waltham, USA) was utilized. Therefore, three samples per each microstructure and blank were immobilized in a 24‐well plate and 1 mL of α‐MEM (Gibco, ThermoFisher Scientific, Waltham, USA), containing 5% fibrinogen‐depleted human platelet lysate (hPLFD, ELAREM, PLBioScience GmbH, Technology Centre Aachen, Germany), and 1% Anti‐Anti (Antibiotic‐Antimycotic, ThermoFisher Scientific, Waltham, USA) were added. They were incubated at 37 °C for 2 h and then washed three times with 1x PBS for 5 min each. To each well, 200 µL of 0.1% Triton X‐100 in distilled water were added and incubated overnight at 4 °C. The following day, 150 µL of each sample were transferred to a 96‐well plate, as well as albumin standards with the following concentrations: 50, 25, 20, 15, 10, 5, 2.5, and 0 µg mL^−1^.

Then, 150 µL of Coomassie Reagent were added to each well and incubated at room temperature for 10 min. The absorbance was measured at 562 nm using a Spark Multimode microplate reader (Tecan group Ltd., Männedorf, Switzerland). The average blank was subtracted from all standards and samples. A standard curve was created to calculate the protein concentration.

### Clinical Specimens

In total, hMSCs isolated from spongiosa samples of ten donors were obtained from the ViBiMeD biobank of the Department of Orthopedics and Traum Surgery (EK‐No. 1822/2017). This biobank includes clinical waste tissue from patients undergoing surgery at the department. All patients had donated their waste tissues for scientific research with written informed consent. The ethics committee of the Medical University of Vienna approved the use of the specimens for the present study (EK‐No. 1138/2024).

### Cell Culture

Isolated primary hMSCs from the ten donors were pooled and cultured in α‐MEM (Gibco, ThermoFisher Scientific, Waltham, USA), containing 5% hPLFD, and 1% Anti‐Anti in a humidified atmosphere of 5% CO_2_ at 37 °C. Upon reaching 80% confluency, hMSCs were passaged up to 4 weeks (4 passages) and used for biocompatibility tests. For assays measuring differentiation potential and mineralization capability, culturing media were replaced with osteogenic differentiation media comprising 200 mм β‐glycerol (Sigma‐Aldrich,Saint Louis, MO, USA), 100 mм ascorbic acid (Sigma‐Aldrich,Saint Louis, MO, USA), and 1 mм dexamethasone (Sigma‐Aldrich,Saint Louis, MO, USA) in α‐MEM full medium (+1% Anti‐Anti, 5% hPLFD).

### Cell Attachment Assay

Initially, hMSCs were resuspended in media, and the concentration was adjusted to 100000 cells ml^−1^. The structured PCL samples underwent a triple cleaning process in a 70% ethanol ultrasonic bath for 5 min. Then, the samples were immobilized in a 24‐well plate. Each sample was covered with 1 ml of the cell suspension and underwent a 2‐h incubation period at 37 °C with 5% CO_2_.

Afterwards, the samples underwent an upside‐down centrifugation at 50 g for five min at room temperature, followed by a three min washing step with 1xPBS. Subsequently, 600 µL of a 1% glutaraldehyde‐PBS solution (Sigma‐Aldrich, Missouri, USA) were applied to each sample and incubated for 15 min. The 1% glutaraldehyde‐PBS solution was removed, and 600 µL of a 0.1% crystal violet solution (Sigma‐Aldrich, Missouri, USA) were added to the samples, shaken, and incubated for 25 min. Following this incubation period, the dye was removed using deionized water.

The previously centrifuged, fixed, and stained samples were analyzed with a Keyence VHX‐7000 digital microscope (Keyence International, Mechelen, Belgium) at 500‐fold magnification. This involved capturing five images at various locations for each sample. Subsequently, the cell count of the stained samples was assessed using Image J.

### Cell Proliferation Evaluation: XTT Viability Assay

Cell viability and proliferation were assessed using the XTT Cell Viability Assay (CyQUANT™, Invitrogen, ThermoFisher Scientific, Waltham, USA) adapted from the manufacturer's instructions to a 24‐well format. In short, three samples per each microstructure and blank were immobilized in a 24‐well plate and 1000 cells were seeded on each sample in a volume of 0.5 mL (2000 cells mL^−1^). The cells were incubated for 1, 3, 7, and 10 days at 37 °C. Three cell‐free wells were used as controls. On the corresponding days, the working solution was prepared by mixing 1 mL of Electron coupling Reagent and 6 mL of tetrazolium‐based compound. Then, 350 µL of the freshly prepared working solution were added to each well. After 4 h of incubation, viability was quantified in terms of absorbance, measured at 450 and 660 nm (reference wavelength) with a Spark Multimode microplate reader (Tecan group Ltd., Männedorf, Switzerland). The specific absorbance was calculated as follows:

(1) $$\begin{array}{ccc} \mathrm{Specific}\;\mathrm{Absorbance} & = & \left[\mathrm{Abs}450\;\mathrm{nm}\left(\mathrm{Test}\right)-\mathrm{Abs}450\;m\left(\mathrm{Blank}\right)\right]\\ & & -\;\mathrm{Abs}660\;\mathrm{nm}\left(\mathrm{Test}\right) \end{array}$$

### Fluorescence Microscopy

To visualize the cytoskeletal configuration of cells that had adhered to the different microstructured surfaces, F‐actin and the nuclei were stained using Actin Green 488 ReadyProbe reagent and NucBlue Fixed Cell Stain ReadyProbes – DAPI special formulation (ThermoFisher scientific, Waltham, USA), respectively.

Initially, 50000 cells mL^−1^ in α‐MEM full medium were seeded onto each microstructured sample and incubated overnight at 37 °C. Subsequently, cells were fixed with 4% formaldehyde prepared in PBS (Fixative solution, Image‐it™ Fix/Perm kit, Invitrogen, ThermoFisher scientific, Waltham, USA) and, after a 15 min incubation time at room temperature, they were washed three times with DPBS (Wash Buffer, Image‐it™ Fix/Perm kit, Invitrogen, ThermoFisher scientific, Waltham, USA). Next, cells were permeabilized by adding 0.5% Triton X‐100 prepared in PBS (Permeabilization solution, Image‐it Fix/Perm kit, Invitrogen, ThermoFisher scientific, Waltham, USA) to each sample and incubating at room temperature for 15 min. After three additional washes with DPBS, the non‐specific binding sites were blocked by incubating the samples in 3% BSA prepared in DPBS (Blocking solution, Image‐it Fix/Perm kit, Invitrogen, ThermoFisher scientific, Waltham, USA) for 60 min at room temperature. The staining procedure involved one mL of DPBS to each sample as a buffer for the dye. ActinGreen 488 ReadyProbes was then added in the amount of two drops and incubated in the dark at room temperature for 15 min, allowing the phalloidin to specifically bind and label the actin filaments within the cells’ cytoskeleton. Subsequently, the nuclei were stained by adding four drops of NucBlue Fixed Cell Stain ReadyProbes – DAPI special formulation to each sample, with an incubation time of 30 min in the dark at room temperature. Finally, the samples were imaged with an Nikon Eclipse Ti confocal microscope (Nikon, Japan) with a 10x objective, and individual pictures were merged using ImageJ.

### Alkaline Phosophatase (ALP) Activity

ALP activity was measured using an Alkaline Phosphatase Assay Kit (Sigma Aldrich, Saint Louis, MO, USA) according to the manufacturers protocol. Briefly, three samples per each microtopography and blank were immobilized in a 24‐well plate and 1000 cells were seeded on each sample in a volume of 0.5 mL (2000 cells mL^−1^). The cells were then incubated in osteogenic differentiation medium for 7 and 14 days, respectively, at 37 °C. At the corresponding days, cells were washed with 1x PBS and lysed with 0.2% Triton X‐100 (SERVA Electrophoresis GmbH, Heidelberg, Germany) by shaking for 20 min at room temperature. 50 µL of sample were transferred into a clear flat bottom 96‐well plate. The working solution was prepared by mixing assay buffer with Mg acetate (0.2 м), and p‐nitrophenyl phosphate (pNPP, 1 м) at a ratio of 200:5:2, at the required volume. 150 µL of this working reagent were added to each well of the 96‐well plate with sample. Additionally, 200 µL of purified water and 200 µL of calibrator were added in triplicates to separate wells of the 96‐well plate. The plate was tapped briefly to mix the solutions. Absorbance was read in 4 min intervals at a wavelength of 405 nm. ALP activity was calculated as given in the manual of the assay.

### Statistical Analysis

For statistical analysis and visual representation of data, GraphPad Prism (V10, GraphPad Software, San Diego, CA, USA) was used. The statistical analysis encompassed the centrifugation adhesion test, contact angle measurements, and protein adsorption, utilizing one‐way ANOVA. The XTT assay and ALP assay were subjected to a two‐way ANOVA. Following ANOVA calculations, post hoc Bonferroni‐corrected t‐tests were performed between individual groups. This correction aimed to mitigate Type I error resulting from multiple testing without excessively diminishing test power. A p‐value less than 0.05 was considered to be statistically significant. Mean values with standard deviation are depicted in the graphs.

### Ethics Approval/Patient Consent Statement

Human mesenchymal stem cells from spongiosa samples of donors were obtained from the ViBiMeD biobank of the Department of Orthopedics and Trauma Surgery (EK‐No. 1822/2017). All patients had donated their waste tissues for scientific research with written informed consent. The ethics committee of the Medical University of Vienna approved the use of the specimens for the present study (EK‐No. 1138/2024).

## Conflict of Interest

The authors declare no conflict of interest.

## Supporting information

Supporting Information

## Data Availability

The data that support the findings of this study are available from the corresponding author upon reasonable request.
