# Supplementary material for: Bone‐Mimetic Osteon Microtopographies on Poly‐ε‐Caprolactone Enhance the Osteogenic Potential of Human Mesenchymal Stem Cells
Source: Macromol Biosci. 2024 Sep 5;25(2):2400311. doi: 10.1002/mabi.202400311 (PMC11827551; doi:10.1002/mabi.202400311)
Supplement: Supplementary file 1 — Supporting Information [file MABI-25-2400311-s001.docx]

Supporting Information

Bone-mimetic Osteon Microtopographies on Poly-ε-caprolactone Enhance the Osteogenic Potential of Human Mesenchymal Stem Cells

Matthias Vostatek, Elettra Verin, Marvin Tamm, Mario Rothbauer, Stefan Toegel, Francesco Moscato*

M. Vostatek, E. Verin, M. Tamm, F. Moscato
Center for Medical Physics and Biomedical Engineering, Medical University of Vienna, Waehringer Guertel 18-20/4L, 1090 Vienna, Austria
E-mail: francesco.moscato@meduniwien.ac.at

M. Vostatek, F. Moscato
Austrian Cluster for Tissue Regeneration, Donaueschingenstrasse 13, 1200 Vienna, Austria

M. Rothbauer, S. Toegel
Karl Chiari Lab for Orthopedic Biology, Department of Orthopedics and Trauma Surgery, Medical University of Vienna, Waehringer Guertel 18-20, 1090 Vienna, Austria

M. Rothbauer
Faculty of Technical Chemistry, Technische Universitaet Wien, Getreidemarkt 9, 1060 Vienna, Austria

M. Rothbauer, S. Toegel
Ludwig Boltzmann Institute for Arthritis and Rehabilitation, Spitalgasse 23/BT88, 1090 Vienna, Austria

F. Moscato
Ludwig Boltzmann Institute for Cardiovascular Research, Waehringer Guertel 18-20/4L, 1090 Vienna, Austria

Experimental Section

To detect calcification ARS was performed. Therefore, three samples per each microtopography and blank were immobilized in a 24-well plate and 50.000were seeded on each sample in a volume of 0.5ml (100.000 cells mL^-1^). The cells were incubated in osteogenic differentiation media for 7 and 14 days respectively at 37°C. At the corresponding days cells were washed with 1x PBS and fixed with 7.5% formaldehyde (SAV Liquid Production GmbH, Germany) for 30 minutes. Followed by another washing step and stained by alizarin red staining solution for 30 minutes, in the dark, at room temperature. The staining solution was aspirated and the samples thoroughly rinsed and stored dry until imaging. Imaging was processed using the Keyence VHX-7000 digital microscope (Keyence International, Mechelen, Belgium).

Results and Discussion

With Alizarin Red staining the calcium deposits after differentiation can be observed in Figure S1. Calcium deposits are displayed on each microtopogrpahy on day 7 and 14. On day 7 the overview image (1x) seems to show that the inverted pyramid structure leads to the most prominent calcium deposition, whereas little red staining on the other substrates. On closer inspection however (500x) calcium deposits can be seen on all microtopography on day 7. On day 14, also the inverted pyramids seem to show the most prominent calcium deposition by vibrant and evenly distributed red staining, followed by osteon-like and grooves, respectively. Blank and protrusion, even though some staining can be seen, seem to have difficulties in calcifying.


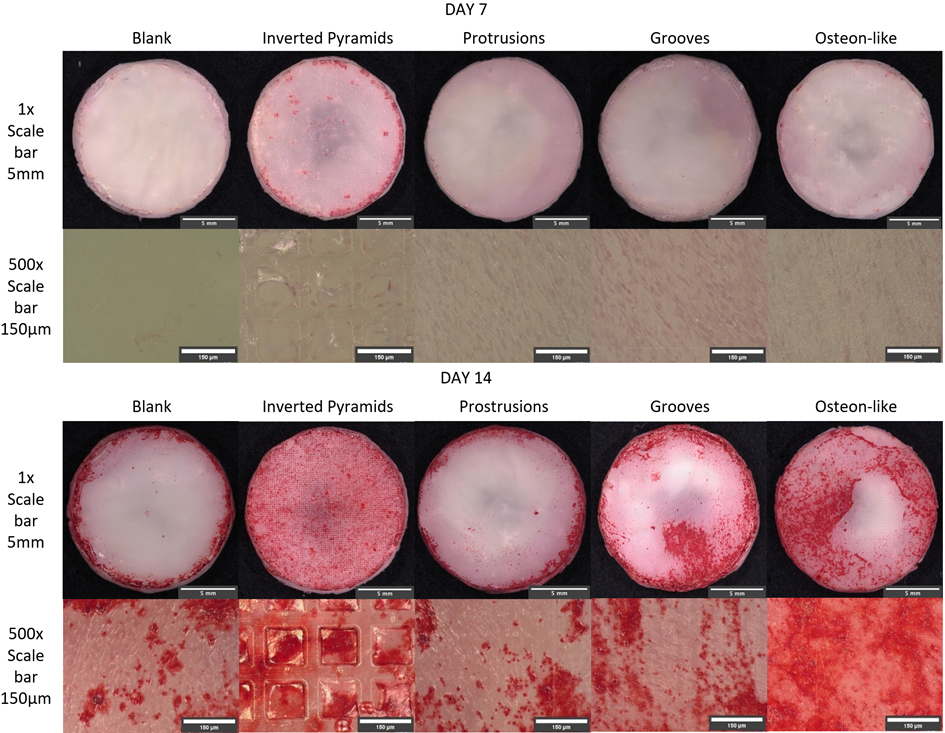


Figure S1: Alizarin Red Staining for calcium deposits on different micropotopgrpahies (blank, inverted pyramids, protrusions, grooves, and osteon-like) at day 7 and 14. 1x of discs with a scale bar of 5mm. 500x with scale bar of 150µm
